# AFRICAN-AMERICAN ALZHEIMER’S CAREGIVER TRAINING AND SUPPORT PROJECT 2 PILOT STUDY: OUTCOMES ANALYSIS

**DOI:** 10.1093/geroni/igac059.2709

**Published:** 2022-12-20

**Authors:** Robert Glueckauf, Nik Lampe, Michelle Kazmer, Alexandra Nowakowski, Yuxia Wang, Naomi Thelusma, Dominique Williams, Cordy McGill-Scarlett

**Affiliations:** Florida State University College of Medicine, Tallahassee, Florida, United States; Vanderbilt University, Nashville, Tennessee, United States; Florida State University, Tallahassee, Florida, United States; Florida State University College of Medicine - Orlando Regional Campus, Orlando, Florida, United States; Florida State University College of Medicine, Tallahassee, Florida, United States; Florida State University College of Medicine, Tallahassee, Florida, United States; Florida State University College of Medicine, Tallahassee, Florida, United States; Florida State University College of Medicine, Tallahassee, Florida, United States

## Abstract

The purpose of this study was to conduct an initial evaluation of the quantitative and qualitative outcomes of the African-American Alzheimer’s Caregiver Training and Support Project 2 (ACTS2). Quantitative objectives focused on assessing changes in caregiver depression and health status, as well as the severity of caregiving and self-care problems from pre- to post-intervention. Secondary quantitative analyses examined post-treatment changes in social support and caregiver burden. Qualitative objectives included examining caregivers’ perceptions of the effectiveness of in-session training activities, quality of relationships among participants and their facilitator, and appraisals of spiritual elements of the program. Nine African American family caregivers of older adults with dementia completed the ACTS2 lay pastoral care facilitator-led, telephone cognitive-behavioral intervention. The twelve-week training program included 7 skills-building groups and 5 individual problem-solving sessions. Significant improvements were found on the majority of dependent measures, including caregiver depression, health status, problem severity, and social support. Qualitative analysis highlighted the value caregivers placed on relationships with their co-participants and group facilitators, the role of spirituality within the program, and the importance of goal setting in improving caregiver distress and self-care. Convergence was found between quantitative and qualitative findings, particularly improvements in the domains of caregiver distress, health status, and social support. Overall, the findings of the pilot study were promising. Replication using a randomized controlled design with a larger sample size is needed to test the reliability of the findings. Benefits of tailoring intervention to caregivers’ sociocultural preferences and spiritual values are also addressed.

